# Supplementary material for: Measuring the success of programmes of care for people living with dementia: a protocol for consensus building with consumers to develop a set of Core Outcome Measures for Improving Care (COM-IC)
Source: BMJ Open. 2023 Dec 9;13(12):e073884. doi: 10.1136/bmjopen-2023-073884 (PMC10729031; doi:10.1136/bmjopen-2023-073884)
Supplement: Author's manuscript [file bmjopen-2023-073884.draft_revisions.pdf]

BMJ Open

BMJ Open is committed to open peer review. As part of this commitment we make the peer review history of every article we publish publicly available.

When an article is published we post the peer reviewers' comments and the authors' responses online. We also post the versions of the paper that were used during peer review. These are the versions that the peer review comments apply to.

The versions of the paper that follow are the versions that were submitted during the peer review process. They are not the versions of record or the final published versions. They should not be cited or distributed as the published version of this manuscript.

BMJ Open is an open access journal and the full, final, typeset and author-corrected version of record of the manuscript is available on our site with no access controls, subscription charges or pay-per-view fees (http://bmjopen.bmj.com).

If you have any questions on BMJ Open's open peer review process please email info.bmjopen@bmj.com

BMJ Open

Measuring the success of programs of care for people living with dementia – a protocol for consensus-building with consumers to develop Core Outcome Measures for Improving Care (COM-IC)

Journal:	*BMJ Open*
Manuscript ID	bmjopen-2023-073884
Article Type:	Protocol
Date Submitted by the Author:	21-Mar-2023

Complete List of Authors:	Comans, Tracy; The University of Queensland, Centre for Health Services Research<br>Nguyen, Kim; The University of Queensland, Centre for Health Service Research; Trinity College, Global Brain Health Institute<br>Gray, Len; The University of Queensland, Centre for Health Services Research<br>Flicker, Leon; The University of Western Australia, WA Centre for Health & Ageing<br>Williamson, Paula; University of Liverpool, Department of Health and Data Science<br>Dodd, Susanna; University of Liverpool, Department of Health and Data Science<br>Kearney, Anna; University of Liverpool, Department of Health and Data Science<br>Cunningham, Colm; University of Sydney, School of Public Heatlh; Hammond Care International<br>Morris, Thomas; University of Sydney, School of Public Health; Hammond Care<br>Nunn, Jack; Science For All; La Trobe University<br>Trepel , Dominic ; Trinity College Dublin, Global Brain Health Institute<br>Almeida, Osvaldo; University of Western Australia, Centre for Health & Ageing<br>Kenny, Danelle; The University of Queensland, Centre for Health Services Research;<br>Welch, Alyssa; The University of Queensland, Centre for Health Services Research<br>Lowthian, Judy; Bolton Clarke, Bolton Clarke Research Institute; Monash University, School of Public Health and Preventive Medicine<br>Quinn, John; Wynnum Manly Dementia Alliance; The University of Queensland, Centre for Health Services Research<br>Petrie, Glenys; Wynnum Manly Dementia Alliance; The University of Queensland, Centre for Health Services Research<br>Dao-Tran, Tiet-Hanh; The University of Queensland, Centre for Health Services Research<br>Manchha, Asmita; Bolton Clarke Research Institute<br>Kurrle, Susan; University of Sydney, School of Public Health

Keywords:	Dementia < NEUROLOGY, Health Services for the Aged, Quality in health

	care < HEALTH SERVICES ADMINISTRATION & MANAGEMENT, Feasibility Studies

SCHOLARONE™
Manuscripts

Title Page

Measuring the success of programs of care for people living with dementia – a protocol for consensus-building with consumers to develop Core Outcome Measures for Improving Care (COM-IC)

Tracy A Comans[1], Kim-Huong Nguyen[1, 2], Len Gray[1], Leon Flicker[3], Paula Williamson[4], Susanna Dodd[4], Anna Kearney[4], Colm Cunningham[5, 6], Thomas Morris[5, 7], Jack Nunn[8,9], Dominic Trepel[2], Osvaldo P. Almeida[3], Danelle Kenny[1], Alyssa Welch[1], Judy Lowthian[10, 11, 12], John Quinn[1, 13], Glenys Petrie[1, 13], Tiet-Hanh Dao-Tran[1], Asmita Manchha[10], Susan Kurrle[5]

Affiliations:

1. Centre for Health Services Research, The University of Queensland, Brisbane, Australia
2. Global Brain Health Institute, Trinity College, Dublin, Ireland
3. WA Centre for Health & Ageing, University of Western Australia, Perth, Australia
4. Department of Health and Data Science, University of Liverpool, Liverpool, United Kingdom
5. School of Public Health, University of Sydney, Sydney, Australia
6. Hammond Care International, London, United Kingdom
7. Hammond Care, Sydney, Australia
8. Science for All, Melbourne, Australia
9. La Trobe University, Melbourne, Australia
10. Bolton Clarke Research Institute, Brisbane, Australia
11. School of Public Health and Preventive Medicine, Monash University, Melbourne, Australia
12. Faculty of Health and Behavioural Sciences, The University of Queensland, Brisbane, Australia
13. Wynnum Manly Dementia Alliance, Manly, Australia

Corresponding author:

Tracy Comans, Level 5 Health Sciences Building, Royal Brisbane and Women's Hospital, Herston. Email: t.comans@uq.edu.au

Keywords: dementia, aged care, outcome assessment, feasibility, standardisation, co-design

Approx word count

Abstract: 200

Main body: 3850

For peer review only

Abstract

Introduction

The Core Outcome Measures for Improving Care (COM-IC) project aims to deliver practical guidelines on the selection and implementation of a suite of core outcomes to measure the effectiveness of interventions for dementia care.

Methods and Analysis

COM-IC embeds a participatory action approach to using the alignment, harmonisation, and results framework for measuring dementia care in Australia. Using this framework, suitable core outcome measures will be identified, analysed, implemented, and audited. The methods for analysing each stage will be co-designed with stakeholders, through the conduit of a stakeholder reference group including people living with dementia, formal and informal carers, aged care industry representatives, researchers, clinicians, and policy actors. The co-designed evaluation methods consider two key factors: feasibility and acceptability. These considerations will be tested during a six-month feasibility study embedded in aged care industry partner organisations.

Ethics and Dissemination

COM-IC has received ethical approval from the University of Queensland (HREC 2021/HE001932).

Results will be disseminated through networks established over the project, and in accordance with both the publication schedule and requests from the stakeholder reference group. Full access to publications and reports will be made available through UQ eSpace (https://espace.library.uq.edu.au/), an open access repository hosted by the University of Queensland.

Article Summary

Strengths and limitations of this study

- A key strength of this study is the use of participatory co-design and co-evaluation methods to embed people with an experience of dementia in the selection of core outcome measures for interventions that affect them.
- This study applies the Alignment, Harmonisation and Results framework to develop and assess feasibility of a set of core outcome measures for dementia care.
- Feasibility will be tested through implementation within partner organisations.
- Limitations are imposed by the short timeframe for the feasibility study.

For peer review only

Introduction

Background and rationale

Dementia is a syndrome characterised by cognitive and functional decline, posing unique and complex challenges for health systems globally (1). Worldwide, there are over 55 million people living with dementia, with significant diversity in symptoms, types, and demographics. Experiences vary widely across many features of the disease, including age of onset, symptoms, severity, duration, and progression. This variability and associated uncertainty demand equal variability in support services and care delivery models, spanning across all care settings and multiple providers.

While the ultimate care objective for all stakeholders is better health and quality of life for care recipients, evaluation models vary widely (2,3). The delivery of interventions and care models across different settings and for different populations, combined with the capability and capacity of service organisations, naturally leads to the use of a wide range of instruments and approaches to report outcomes and to measure success (clinical efficacy and effectiveness, care quality and efficiency), despite their shared focus on dementia. The use of different assessment instruments reduces the comparability of results across care models, leading to the slow translation of evidence into practice and reduced capacity to improve quality of care. It also results in fragmentation and duplication of service provision across providers, creating system-wide inefficiencies.

There is a consensus among stakeholder groups that inefficiencies in dementia care should be minimised, and that meaningful outcome data that are consistent, comparable, and feasible, are crucial to improving quality of care (4,5). The Core Outcome Measures for Improving Care (COM-IC) project is aligned with several recommendations made in 2021 by the Royal Commission into Aged care Quality and Safety. Specifically, the project aligns with: requirements for ongoing research into the development and use of an evidence base for quality indicators; publication and guidance on data use; methodology for benchmarking; setting improvement targets; and public reporting of performance against measurable data (6).

Involving stakeholders in the design and development of research further enhances quality improvement by increasing the relevance of selected measures to people affected by dementia (7). Stakeholders are identified in this project as anyone affected by dementia, including people living with dementia, their formal and informal carers, family and friends, aged care organisations, advocacy groups, health professionals and government representatives. Statements from the World Health Organisation (WHO) proclaim that people with lived experience of dementia have the greatest direct interest in research outputs and are therefore central to the research process and should be involved in all stages (1). Involving people in research means conducting research with people, as opposed to 'on' or 'for' people (8). People with cognitive impairment are capable of contributing equally to decisions about their care and have the right to voice their preferences. This capability extends beyond individual care planning into decisions about policy and research priorities, and individuals are generally positive about engaging in tasks despite difficulties imposed by their condition (9). The involvement of people affected by dementia in the identification of core outcomes to measure the quality of their care creates a balance between individual autonomy and collective community values that facilitates equitable access to health and care services (7,10,11).

The aim of this protocol is to outline the research plan for COM-IC. This encompasses the development of a process for involving people living with dementia in decisions about the measurement of dementia care, using participatory methods to facilitate working partnerships with stakeholders, enabling timely translation of evidence-based research into practice.

For peer review only

Methods and analysis

Study setting

The majority of people living with dementia seeking care access services in the aged care setting, either in residential care, or in community-based aged care programs (9,12). As such, the COM-IC project is positioned in the aged care setting, incorporating both residential aged care and home care services.

Design

The research design is a multi-component study that applies the Alignment – Harmonisation – Results (AHR) framework (13) that preferences stakeholder engagement through Participatory Action Research (PAR) methodology (14).

Alignment – Harmonisation – Results Framework
The AHR Framework is a model developed to improve resource allocation in global aid, a system with similar complexities to health care quality (13). The COM-IC project adapts this framework to fit the dementia care context (Figure 1).

Figure 1 AHR Framework

Alignment
Alignment is achieved through reaching agreement on a suite of standardised (core) outcomes that define a baseline or minimum standard for all dementia-related interventions and care delivery models in Australia. The alignment phase begins with a review of international core outcome sets for dementia, using the COMET (Core Outcome Measures in Effectiveness Trials) database, including their application and use (3,15,16). Industry partners will then provide information on dementia outcome measures currently used in the Australian context, and the Stakeholder Reference Group (SRG) will develop a consensus on stakeholder preferences (17). A gap analysis will converge these three viewpoints, providing clarity around outcome measures that are relevant to the provision of high quality dementia care, and are feasible to implement in the Australian context (2).

Identified outcome measures with high relevance to people living with dementia will be assessed for methodological quality and relevance to the population and setting (Table 1).

Table 1 Criteria for inclusion of outcome measures

Criterion	Description
**Clarity**	An indicator should be specific, stating clearly what is being measured, how it is measured and its interpretation
**Validity**	The indicator measures what it intends to measure (i.e., it discriminates between good and bad performance), is not subject to large variation due to random changes in small numbers of events, and minimises potential bias
**Measurability and timeliness**	The performance aspect should be measurable and quantifiable
**Consistency over time**	The indicator should measure quality aspects consistently over time and in a timely manner
**Added value**	The indicator should capture elements of performance not captured elsewhere

For peer review only

**Measure of value (benefit)**	The indicator should capture aspects of performance proven to be valued by users and providers of services
**Attribution and granularity**	The indicator should measure aspects of performance that can be attributed to healthcare or aged care settings, and that are subject to healthcare or aged care system control
**Ease of collection**	The indicator must be able to be collected within usual practice in settings where it is required to be collected or minimise additional burden on collectors if this is not possible.

Notes: modified from the Accounting for the Quality of NHS Output framework (18)

Subsequently, this information will be summarised in plain English as a decision-making tool for the Stakeholder Reference Group (SRG). A modified e-Delphi method will be applied to reach consensus, using the STARDIT tool as the primary reporting mechanism (17,19,20). This approach uses structured communication techniques applied systematically to stakeholder panels through several rounds (internet-based surveys) until consensus is reached (20). Each round is summarised by the group facilitator and results reported to all participants before the next round. Rounds continue until consensus is achieved and the measures with consensus will become the core outcome measures recommended in the guidelines. Consensus criteria are modified from Accounting for the Quality of NHS Output (18), being: clarity; validity; measurability and timeliness; consistency over time; added value; measure of value; attribution and granularity; and ease of collection.

Harmonisation

Harmonisation refers to the generation of a standardised set of guidelines for core outcome measures in dementia care. Following the GRADE method, these guidelines will include methods to appraise, monitor and evaluate interventions and models of care (e.g. functional decline, economic evaluation, societal benefit cost analyses), or methods to measure (key) performance indicators at the organisation and system levels (e.g. composite indicators for quality of care and operational efficiency at the organisation level, or equality/disparity of access and utilisation at the sector level) (21). They will also include guidance for the selection of core outcomes for well-defined target populations (e.g. people with younger onset dementia), types of interventions, care settings and sectors. The guidelines will be accompanied by a recommended implementation plan that addresses potential barriers to implementation identified through the Alignment phase, a clear timeline, and stakeholder responsibilities.

A Guideline Development Group (GDG) will be established that comprises members of the research team, SRG, and representatives from the Australian Department of Health and Aged Care.

The Harmonisation phase will also include preparation for the feasibility study (to be conducted in the Results phase). Outcomes of the gap analysis will be used to identify data that are not currently collected by partner organisations. The feasibility of data collection will be determined through focus groups and interviews conducted internally with partner organisations' staff, care recipients and family carers to understand whether additional data collection is acceptable, and the most practical methods of collection. An adoption timeline will be developed, and a sub-set of outcome measures identified that are deemed feasible for trial implementation within the partner organisation.

Results

The results phase will comprise a feasibility study with industry partners that focuses on the translation of research into practice. Data relevant to core outcome measures will be extracted from partner organisations' databases pre- and post-study and evaluated to determine both the functionality and usability of the outcome measures. A qualitative evaluation of the implementation experience will determine the practicality and sustainability of the guidelines and implementation plan developed in the harmonisation phase.

For peer review only

Each partner organisation will trial the implementation of guideline-recommended measures that can be collected within existing data collection systems for six months. This may include modification of existing instruments to collect additional information relating to new recommended indicators.

The guidelines and implementation plan will be co-evaluated by the SRG and investigation team, and revised based on outcomes of the feasibility study (22). Any identified corrections will be made to produce a final set of recommendations.

PPI Statement (Stakeholder Engagement)

Methods used to involve people affected by dementia in this project have been informed by the participatory action research (PAR) paradigm (23). This approach was selected following consultation with people with lived experience of dementia. The PAR paradigm guides stakeholder involvement alongside the AHR framework by embedding participation and action in the research process. Involvement refers to using participatory methods such as workshops, focus groups, Delphi panels, surveys, policy exercises, and mutual learning activities to facilitate inclusion of stakeholders with previously limited involvement in research activities like development, design, conduct, analysis, interpretation, dissemination, implementation, and evaluation (14,24).

Population and participation

The potential involvement of people with cognitive impairment carries additional ethical considerations (25,26). Involvement of people living with dementia is actively encouraged and supported to maximise inclusivity, with levels of influence and support tailored to individual capabilities. People with a lived experience of dementia will be involved with all aspects of research design and implementation. The establishment and operation of the Stakeholder Reference Group will be recorded and analysed to demonstrate the changes in the wider research team and the influence of PAR on final research recommendations. Evidence of personal development over the project will be collected through the dynamic learning needs assessment, a quarterly survey completed by all members of the SRG, investigator team and project team. Evidence of impact will be collected through documentation of meetings and correspondence between consumer participants and the investigation team. Methods of involving people, reported personal development, and other impacts and outcomes will be reported using STARDIT(19).

Stakeholder Reference Group (SRG)

The SRG will guide participatory action throughout the AHR phases. The main role of the SRG is to ensure the measures including in the COM-IC guidelines are meaningful and relevant to people living with dementia while being practical and sustainable for care providers. The SRG will include a diverse range of stakeholders that reflects the heterogeneity of different groups affected by dementia.

Eligibility

The SRG will have between eight and 15 members, including at least four people who have either had an experience of using dementia care and services in the last 10 years, or have been affected by dementia (including parents, loved ones and care partners). The SRG will also include at least one person who identifies as Aboriginal or Torres Strait Islander (with or without dementia), at least two people with professional dementia research or health professional experience, and at least two people with a professional experience of working in dementia care. SRG members will be expected to speak from their personal experience and, where appropriate, collate the views of people with an experience of dementia. SRG members will require a working knowledge of basic computer software – training will be provided as required to support participation.

Recruitment and participation

For peer review only

Members of the SRG will be recruited through an online expression of interest, promoted through the professional and industry networks of the research team and established dementia support networks. Expressions of interest will be reviewed by a subset of the research team, including two consumer investigators. Members will be selected based on their experience, ability to represent a diverse population, and the time they are able to commit to SRG activities. Once informed consent is received from all SRG members, and any competing or conflicting interests have been declared and publicly reported, SRG activities will commence. Potential SRG members must have sufficient cognitive capacity at the time of their recruitment to provide informed consent – individuals incapable of providing consent will not be invited to join the SRG. Potential SRG members with a diagnosis of dementia or cognitive impairment will be contacted by a member of the investigator team with experience working with people living with dementia, who will discuss what SRG participation involves, address any concerns, and confirm informed consent to participate. Acknowledging that dementia is a degenerative condition with variable rates of decline in cognitive capacity, consent will be confirmed and assented at each project activity.

Working groups

Task-specific working groups may be established to seek broader input and experiences at some stages of the COM-IC project. An online expression of interest process similar to that for the SRG will be used to identify participants for each working group. Depending on the activity's objectives, eligibility criteria may include specific research experience, experience of living with dementia and/or experience of providing informal or formal care for a person living with dementia.

Outcome measures

Primary outcome

The primary outcome is the creation of guidelines for a recommended standard set of outcome measures for routine care of people living with dementia, tested for acceptability and feasibility in the Australian context.

Secondary outcomes

Secondary outcomes will include:

- Effectiveness of the PAR methodology, including recruitment, functioning of the stakeholder reference group and ability to achieve consensus, measured using information such as meeting attendance, participation in activities, contributions, fulfilled roles, and surveys.
- Changes in stakeholder and researcher capacity, perspectives and beliefs measured using a dynamic Learning Needs Analysis. All researchers and SRG members will complete the survey quarterly, monitoring changes in attitudes, skills, and needs, with a view to address any identified skills shortages as they become known. This analysis also provides a foundation for preference-mapping and skill-matching to research activities, enabling skilled members to mentor and train those with identified need.

Data collection

Data will be collected by members of the investigation team and industry partners. Data collected by the investigator team will include information about the PAR process, SRG and working group participants and activities. Data collected by industry partners will include information related to the feasibility study (outcome measures, implementation, acceptability). All data collection will be electronic.

Data management

For peer review only - http://bmjopen.bmj.com/site/about/guidelines.xhtml

Data collected for the COM-IC Project will be managed by the project team at The University of Queensland. Data will be protected by multi-factor authentication across two platforms; Microsoft Teams, and the secure research data manager (RDM) developed by the University of Queensland and cited as an approach to good research data management practice (27). All data will be regularly backed-up as part of the RDM. Password protection and multi-factor authorisation will be used to ensure that only approved members of the research and project teams will be able to access data. Any identifiable data will be de-identified prior to any public dissemination. Any information collected by partner organisations will be securely stored under password and two-factor authorisation until it can be de-identified and transferred to The University of Queensland.

Quality control

The secretariat will routinely evaluate data collection and storage and maintain the RDM record. Any adjustments required will be communicated with the group and regular data reports presented at quarterly meetings.

Analysis plan

The final data analysis plan will be co-developed with the SRG and is dependent on the outcome measures identified during guideline development. Data analysis will be jointly conducted by the research team, SRG, and working groups, using agreed methods appropriate to data type. Stakeholders will be involved in data analysis and checking, developing conclusions, and reporting outcomes. Any conclusions from the analysis will be collectively agreed and disseminated in a publicly accessible way, including open-access, peer-reviewed publications. Analysis will focus on determining the feasibility of implementing core outcome measures for dementia care, the impact and effectiveness of PAR, and the data collected by the core outcomes set.

Feasibility analysis will be jointly conducted by the investigator team and a feasibility working group that includes members of the SRG. The methodology includes tools such as surveys and interviews to measure change readiness, beliefs, attitudes, and behaviours of system actors that influence implementation, measures of fidelity, and time and cost of guideline implementation.

Impact and effectiveness of PAR will be analysed using data on *level of engagement* (e.g. meeting attendance, participation in activities, contributions, and roles undertaken), *change in research capacity* (based on the dynamic learning needs assessment), and *ability to achieve consensus (*using tools such as a modified e-Delphi method). The overall PAR methodology will be reported using STARDIT (19). SRG and working group impact on the COM-IC Project will be evaluated using qualitative methods, including data mapping and familiarisation; transcription; coding; searching for themes; reviewing themes with study team members; labelling and summarising themes; and reporting the findings. In order to enhance validity of the analysis, two study team members will independently analyse the data thematically, with the analysis then checked for validity ('member checked') by a third study team member (28–30). STARDIT-PM categories will be used to organise data into pre-defined 'super-categories' that allow consistent comparison with other initiatives reporting with STARDIT(19).

Core outcome set data analysis methods will be co-developed by the SRG and investigator team and will incorporate suitable quantitative methods, e.g., descriptive statistics such as mean, standard deviation, interquartile range and baseline comparisons.

Discussion

Improving care for people living with dementia requires timely and reliable diagnosis, empowerment, and re-enablement of each individual in participating and planning their care, speedy translation of

For peer review only

clinical evidence into practice and policy, and effective co-ordination of care provision across settings. Rapid translation of evidence into research remains a challenge due to the complexity of healthcare and social care services, limited funding resources for services, and lack of consensus on what constitutes "good value care" between individuals living with dementia, service providers and funders.

Rapid translation of COM-IC project findings is feasible as many outcome measures are already embedded in existing information systems and used as key performance indicators or accreditation standards by aged care providers (31,32), and/or used in dementia research (trials, cohort or implementation studies of innovative care delivery models) (4,16,33–35).

The outcome of this project will be guidelines for a recommended set of core outcome measures for routine care of people living with dementia that encompass both consumer preferences and international evidence-based care and are tested and suitable for use in the aged care setting for people living with dementia.

Ethics and dissemination

Research ethics approval

This study has received ethical approval from the University of Queensland (HREC 2021/HE001932) and has received approval in-kind from partner organisations. Any modifications to the protocol that may impact on the conduct of the study, including changes of study objectives, study design, study population, sample sizes, study procedures, or significant administrative aspects will be formalised as a protocol amendment, subject to HREC approval and subsequent notifications to authorities in accordance with local regulations.

Additional considerations for potential cognitive impairment

Accommodations for people with varying levels of cognitive ability, including the potential for deterioration over the course of the study, have been discussed. In the '*Blueprint for dementia research*', the WHO highlights the importance of involving people with lived experience at all stages of the research process, noting it is part of their rights under United Nations Convention on the Rights of Persons with Disabilities and other human rights instruments (1). Involvement ensures the most efficient translation of research into practice, producing outcomes that are relevant to the people they most affect.

In line with the WHO blueprint, additional consideration has been given for accessibility, data-sharing, capacity building, strengthening support networks, utilising existing advocacy and support structures, use of technology, and sharing knowledge (1). Information is packaged in different ways and able to be tailored to the level of comprehension of the individual. Ongoing dynamic learning needs assessment monitors progress and motivation within the project team. Outcomes of the research will be published and distributed widely along familiar routes including academic and industry publications, symposia, and conference presentations. Audiences are diverse, including people with lived experience, clinicians, care professionals, aged care providers, advocacy groups and academics. Assent processes are used to confirm ongoing understanding of the research process. Representation of people with lived experience at every stage is factored into the design, as well as strategies for addressing any discomfort or potential harm as it is identified. Once the SRG is established, members will distribute findings through their networks and recruit working-group members from the dementia community. Any findings will be distributed through SRG networks, meaning the broader dementia community have rapid access to results and have a familiar conduit to provide feedback in real-time to the research team over the course of the project.

For peer review only

Consent and assent

Prior to commencing any research activities, all people included in the study will receive written participant information sheet and will sign a consent form. They will have the opportunity to ask any questions. For people with potential cognitive impairment, pictorial versions have been developed. As dementia is a progressive condition with uncertain prognosis, assent is requested prior to any recording of information. Participants are not required to participate against their will and are free to withdraw at any time.

Confidentiality

All potentially sensitive information will be stored securely on The University of Queensland's research data management system (RDM). This system complies with all international data safety requirements. All project-related data will be stored in a durable format alongside project metadata, which will be regularly backed up by secure ITS servers. Data is accessible only to individuals listed on the project record and are accessible only via institutional usernames and passwords. Information published in reports, academic papers, and presentations, will be de-identified. There is a small risk of identification through familiarity explained to participants in the consent process.

Dissemination of research findings

Results will be disseminated through networks established over the project, and in accordance with both the publication schedule and STARDIT reports.

Data Statement

Full access to outputs will be made available through UQ eSpace, an open access repository hosted by the University of Queensland.

Funding statement

This research is funded by a Medical Research Future Fund (Dementia Ageing and Aged Care Mission) targeted grant, awarded in 2021 (2007650). The funder has no role in study design, data collection, analysis or publications decisions.

Competing interests

Jack Nunn is the volunteer Director of the registered charity 'Science for All,' and is paid as an individual by the charity for his work on this MRFF funded project by written agreement. Science for All will receive funding through the COM-IC MRFF grant to conduct elements of the research program. All funding is reported annually through the Australian Charities and No-for-profits Commission (ACNC).

For peer review only

For peer review only

Authors Contributions

Concept: Developed through collaboration and communication between TC, KHN, LG, LF, PW, SD, CC, TM, JN, DT, OA, AW, JL, JQ, GP and SK.

Design: All authors contributed design ideas, finalised by TC, AW, DK.

Component leads: Overall project: TC; International review of core outcome measures led by AK; guideline development led by TC and SK; Industry analysis led by TM and AM, supported by CC and JL; Gap analysis conducted by THDT, assisted by DK, AK, TM and AM. Guideline development: SK and TC; Feasibility study; JL, CC, supported by AM and TM.Stakeholder Reference Group: JN supported by DK.

Industry Contributions: LF, OA represent hospital care; CC, TM, JL, AM represent residential aged care and home care for older people; CC, SK, LF represent advocacy and dementia support networks. DT, KHN, AW, SD, PW give expertise and insight on core outcome measures and their applications.

Stakeholder Reference Group establishment and analysis; JN, DK, AW, TC.

Writing: TC, DK, AW.

Editing: All authors assist with editing and proofreading manuscript for final submission.

For peer review only

References

1. World Health Organisation. A blueprint for dementia research [Internet]. Creative Commons; 2022. Available from: https://www.who.int/publications/i/item/9789240058248

2. Lord K, Beresford-Dent J, Rapaport P, Burton A, Leverton M, Walters K, et al. Developing the New Interventions for independence in Dementia Study (NIDUS) theoretical model for supporting people to live well with dementia at home for longer: a systematic review of theoretical models and Randomised Controlled Trial evidence. Soc Psychiatry Psychiatr Epidemiol. 2020 Jan 1;55(1):1–14.

3. Williamson PR, Altman DG, Bagley H, Barnes KL, Blazeby JM, Brookes ST, et al. The COMET Handbook: version 1.0. Trials. 2017 Jun 20;18(Suppl 3):280.

4. Dodd S, Harman N, Taske N, Minchin M, Tan T, Williamson PR. Core outcome sets through the healthcare ecosystem: the case of type 2 diabetes mellitus. Trials. 2020 Jun 25;21:570.

5. Tran A, Nguyen KH, Gray L, Comans T. A Systematic Literature Review of Efficiency Measurement in Nursing Homes. Int J Environ Res Public Health. 2019 Jun;16(12):2186.

6. Aged Care Royal Commission. Royal Commission into Aged Care Final Report [Internet]. Royal Commission into Aged Care Quality and Safety. [cited 2022 May 24]. Available from: https://agedcare.royalcommission.gov.au/publications/final-report

7. Bethell J, Pringle D, Chambers LW, Cohen C, Commisso E, Cowan K, et al. Patient and Public Involvement in Identifying Dementia Research Priorities. J Am Geriatr Soc. 2018;66(8):1608–12.

8. Briefing notes for researchers - public involvement in NHS, health and social care research [Internet]. [cited 2023 Jan 7]. Available from: https://www.nihr.ac.uk/documents/briefing-notes-for-researchers-public-involvement-in-nhs-health-and-social-care-research/27371

9. Waller M, Mishra GD, Dobson AJ. Estimating the prevalence of dementia using multiple linked administrative health records and capture–recapture methodology. Emerg Themes Epidemiol. 2017 Feb 27;14:3.

10. Kelly S, Lafortune L, Hart N, Cowan K, Fenton M, Brayne C. Dementia priority setting partnership with the James Lind Alliance: using patient and public involvement and the evidence base to inform the research agenda. Age Ageing. 2015 Nov;44(6):985–93.

11. Ratcliffe J, Hutchinson C, Milte R, Nguyen KH, Welch A, Caporale T, et al. How do people with dementia and family carers value dementia-specific quality of life states? An explorative “Think Aloud” study. Australas J Ageing. 2019;38(S2):75–82.

12. Rozsa M, Ford A, Flicker L. Assessing people with dementia: the role of the aged care assessment team and memory clinics. Intern Med J. 2016;46(9):1081–8.

13. OECD World Bank. Harmonisation, Alignment, Results [Internet]. The World Bank; 2007. Available from: https://doi.org/10.1596/978-9-2640-3673-4

14. NIHR. What is Patient and Public Involvement and Engagement? - HPRUEZI [Internet]. [cited 2023 Jan 17]. Available from: http://www.hpruezi.nihr.ac.uk/public-involvement/

15. Page MJ, Moher D, Bossuyt PM, Boutron I, Hoffmann TC, Mulrow CD, et al. PRISMA 2020 explanation and elaboration: updated guidance and exemplars for reporting systematic reviews. BMJ. 2021 Mar 29;n160.

16. COMET. COMET Initiative | Home [Internet]. [cited 2022 May 24]. Available from: https://www.comet-initiative.org/

For peer review only

17. Lee A, Davies A, Young A. Systematic review of international Delphi surveys for core outcome set development: representation of international patients. BMJ Open. 2020 Nov 23;10:e040223-undefined.

18. Bojke C, Castelli A, Grasic K, Mason A, Street A. Accounting for the quality of NHS output [Internet]. Working Papers. Centre for Health Economics, University of York; 2018 Apr [cited 2022 Aug 25]. (Working Papers). Report No.: 153cherp. Available from: https://ideas.repec.org/p/chy/respap/153cherp.html

19. Nunn JS, Shafee T, Chang S, Stephens R, Elliott J, Oliver S, et al. Standardised data on initiatives—STARDIT: Beta version. Res Involv Engagem. 2022 Dec;8(1):31.

20. Hasson F, Keeney S, McKenna H. Research guidelines for the Delphi survey technique. J Adv Nurs. 2000;32(4):1008–15.

21. Cochrane. GRADE handbook [Internet]. [cited 2022 May 24]. Available from: https://gdt.gradepro.org/app/handbook/handbook.html

22. Phillips R, Phillips K, Phillips H. Creative Writing for Social Research: A Practical Guide [Internet]. Policy Press; 2021 [cited 2023 Jan 17]. Available from: https://bristoluniversitypressdigital.com/view/book/9781447355991/9781447355991.xml

23. Pandya-Wood R, Barron DS, Elliott J. A framework for public involvement at the design stage of NHS health and social care research: time to develop ethically conscious standards. Res Involv Engagem. 2017 Dec;3(1):6.

24. INVOLVE. Public involvement in research: values and principles framework [Internet]. INVOLVE: Eastleigh; 2015 [cited 2021 Feb 26]. Available from: https://www.invo.org.uk/posttypepublication/public-involvement-in-researchvalues-and-principles-framework/

25. Chandra M, Harbishettar V, Sawhney H, Amanullah S. Ethical Issues in Dementia Research. Indian J Psychol Med. 2021 Sep;43(5 Suppl):S25–30.

26. NHMRC. National Statement on Ethical Conduct in Human Research (2007) (Updated May 2015) | National Health and Medical Research Council [Internet]. 2011 [cited 2017 Nov 10]. Available from: https://www.nhmrc.gov.au/book/national-statement-ethical-conduct-human-research

27. Directorate-General for Research and Innovation (European Commission). Turning FAIR into reality: final report and action plan from the European Commission expert group on FAIR data [Internet]. LU: Publications Office of the European Union; 2018 [cited 2023 Jan 10]. Available from: https://data.europa.eu/doi/10.2777/1524

28. Saldaña J. The coding manual for qualitative researchers. 3E [Third edition]. Los Angeles ; London: SAGE; 2016. 339 p.

29. Hyett N, Kenny A, Dickson-Swift V. Methodology or method? A critical review of qualitative case study reports. Int J Qual Stud Health Well-Being. 2014 Jan 1;9(1):23606.

30. Guest G, MacQueen K, Namey E. Applied Thematic Analysis [Internet]. 2455 Teller Road, Thousand Oaks California 91320 United States: SAGE Publications, Inc.; 2012 [cited 2023 Jan 8]. Available from: https://methods.sagepub.com/book/applied-thematic-analysis

31. Comans T, Nguyen K, Zelenyuk V, Gray L, Tran A, Nguyen B, et al. Research Paper 9 - The cost of residential aged care [Internet]. Royal Commission into Aged Care Quality and Safety. [cited 2021 Feb 26]. Available from: https://agedcare.royalcommission.gov.au/publications/research-paper-9-cost-residential-aged-care

32. Tran A, Nguyen KH, Gray L, Comans T. A Systematic Literature Review of Efficiency Measurement in Nursing Homes. Int J Environ Res Public Health. 2019 Jan;16(12):2186.

For peer review only

33. Dodd S, Clarke M, Becker L, Mavergames C, Fish R, Williamson PR. A taxonomy has been developed for outcomes in medical research to help improve knowledge discovery. J Clin Epidemiol. 2018 Apr;96:84–92.

34. Vincent R, Chalmers J r., McWilliams C, Thomas K s., Dodd S, Rogers N, et al. Assessing uptake of the Harmonising Outcome Measures for Eczema (HOME) Core Outcome Set and recommended instruments. Br J Dermatol. 2020;183(3):566–8.

35. Comans T, Nguyen K, Ratcliffe J, Rowen D, Mulhern B. Valuing the AD-5D Dementia Utility Instrument: An Estimation of a General Population Tariff. PharmacoEconomics. 2020 Apr 21;38:871–81.

For peer review only

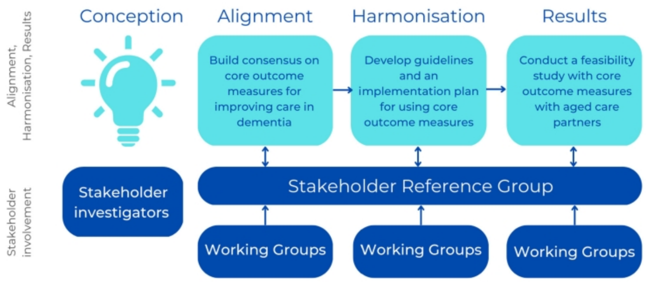

Figure 1: AHR Framework

210x297mm (300 x 300 DPI)

BMJ Open

Measuring the success of programs of care for people living with dementia – a protocol for consensus-building with consumers to develop a Core Outcome Set for Improving Care (COM-IC)

Journal:	*BMJ Open*
Manuscript ID	bmjopen-2023-073884.R1
Article Type:	Protocol
Date Submitted by the Author:	14-Oct-2023
Complete List of Authors:	Comans, Tracy; The University of Queensland, Centre for Health Services Research<br>Nguyen, Kim; The University of Queensland, Centre for Health Service Research; Trinity College, Global Brain Health Institute<br>Gray, Len; The University of Queensland, Centre for Health Services Research<br>Flicker, Leon; The University of Western Australia, WA Centre for Health & Ageing<br>Williamson, Paula; University of Liverpool, Department of Health and Data Science<br>Dodd, Susanna; University of Liverpool, Department of Health and Data Science<br>Kearney, Anna; University of Liverpool, Department of Health and Data Science<br>Cunningham, Colm; University of Sydney, School of Public Heatlh; Hammond Care International<br>Morris, Thomas; University of Sydney, School of Public Health; Hammond Care<br>Nunn, Jack; Science For All; La Trobe University<br>Trepel , Dominic ; Trinity College Dublin, Global Brain Health Institute<br>Almeida, Osvaldo; University of Western Australia, Centre for Health & Ageing<br>Kenny, Danelle; The University of Queensland, Centre for Health Services Research;<br>Welch, Alyssa; The University of Queensland, Centre for Health Services Research<br>Lowthian, Judy; Bolton Clarke, Bolton Clarke Research Institute; Monash University, School of Public Health and Preventive Medicine<br>Quinn, John; Wynnum Manly Dementia Alliance; The University of Queensland, Centre for Health Services Research<br>Petrie, Glenys; Wynnum Manly Dementia Alliance; The University of Queensland, Centre for Health Services Research<br>Dao-Tran, Tiet-Hanh; The University of Queensland, Centre for Health Services Research<br>Manchha, Asmita; Bolton Clarke Research Institute<br>Kurrle, Susan; University of Sydney, School of Public Health
Primary Subject	Health economics

Heading:	
Secondary Subject Heading:	Health services research
Keywords:	Health Services for the Aged, Quality in health care < HEALTH SERVICES ADMINISTRATION & MANAGEMENT, Feasibility Studies, Dementia

SCHOLARONE™
Manuscripts

Title Page

Measuring the success of programs of care for people living with dementia – a protocol for consensus-building with consumers to develop a Core Outcome Set for Improving Care (COM-IC)

Tracy A Comans[1], Kim-Huong Nguyen[1, 2], Len Gray[1], Leon Flicker[3], Paula Williamson[4], Susanna Dodd[4], Anna Kearney[4], Colm Cunningham[5, 6], Thomas Morris[5, 7], Jack Nunn[8,9], Dominic Trepel[2], Osvaldo P. Almeida[3], Danelle Kenny[1], Alyssa Welch[1], Judy Lowthian[10, 11, 12], John Quinn[1, 13], Glenys Petrie[1, 13], Tiet-Hanh Dao-Tran[1], Asmita Manchha[10], Susan Kurrle[5]

Affiliations:

1. Centre for Health Services Research, The University of Queensland, Brisbane, Australia
2. Global Brain Health Institute, Trinity College, Dublin, Ireland
3. WA Centre for Health & Ageing, University of Western Australia, Perth, Australia
4. Department of Health and Data Science, University of Liverpool, Liverpool, United Kingdom
5. School of Public Health, University of Sydney, Sydney, Australia
6. Hammond Care International, London, United Kingdom
7. Hammond Care, Sydney, Australia
8. Science for All, Melbourne, Australia
9. La Trobe University, Melbourne, Australia
10. Bolton Clarke Research Institute, Brisbane, Australia
11. School of Public Health and Preventive Medicine, Monash University, Melbourne, Australia
12. Faculty of Health and Behavioural Sciences, The University of Queensland, Brisbane, Australia
13. Wynnum Manly Dementia Alliance, Manly, Australia

Corresponding author:

Tracy Comans, Level 5 Health Sciences Building, Royal Brisbane and Women's Hospital, Herston.
Email: t.comans@uq.edu.au

Keywords: dementia, aged care, outcome assessment, feasibility, standardisation, co-design

Approx word count

Abstract: 200

Main body: 3850

For peer review only

Abstract

Introduction

The Core Outcome Measures for Improving Care (COM-IC) project aims to deliver practical recommendations on the selection and implementation of a suite of core outcomes to measure the effectiveness of interventions for dementia care.

Methods and Analysis

COM-IC embeds a participatory action approach to using the alignment, harmonisation, and results framework for measuring dementia care in Australia. Using this framework, suitable core outcome measures will be identified, analysed, implemented, and audited. The methods for analysing each stage will be co-designed with stakeholders, through the conduit of a stakeholder reference group including people living with dementia, formal and informal carers, aged care industry representatives, researchers, clinicians, and policy actors. The co-designed evaluation methods consider two key factors: feasibility and acceptability. These considerations will be tested during a six-month feasibility study embedded in aged care industry partner organisations.

Ethics and Dissemination

COM-IC has received ethical approval from the University of Queensland (HREC 2021/HE001932).

Results will be disseminated through networks established over the project, and in accordance with both the publication schedule and requests from the stakeholder reference group. Full access to publications and reports will be made available through UQ eSpace (https://espace.library.uq.edu.au/), an open access repository hosted by the University of Queensland.

Article Summary

Strengths and limitations of this study

- A key strength of this study is the use of participatory co-design and co-evaluation methods to embed people with an experience of dementia in the selection of core outcome measures for interventions that affect them.
- This study applies the Alignment, Harmonisation and Results framework to develop and assess feasibility of a set of core outcome measures for dementia care.
- Feasibility will be tested through implementation within partner organisations.
- Limitations are imposed by the short timeframe for the feasibility study.

For peer review only

Introduction

Background and rationale

Dementia is a syndrome characterised by cognitive and functional decline, posing unique and complex challenges for health systems globally (1). Worldwide, there are over 55 million people living with dementia, with significant diversity in symptoms, types, and demographics. Experiences vary widely across many features of the disease, including age of onset, symptoms, severity, duration, and progression. This variability and associated uncertainty demand equal variability in support services and care delivery models, spanning across all care settings and multiple providers. Under these conditions, evaluation models vary widely (2,3) and it is increasingly difficult to compare interventions and ascertain the value and impact on quality of care of one intervention in relation to another.

The delivery of interventions and care models across different settings and for different populations, combined with the capability and capacity of service organisations, naturally leads to the use of a wide range of instruments and approaches to report outcomes and to measure success (clinical efficacy and effectiveness, care quality and efficiency), despite their shared focus on dementia. The use of different assessment instruments reduces the comparability of results across care models, leading to the slow translation of evidence into practice and reduced capacity to improve quality of care. It also results in fragmentation and duplication of service provision across providers, creating system-wide inefficiencies. In Australia, there is no recommendation or mandatory reporting of outcomes relating specifically to the provision or quality of care for people living with dementia, despite the increasing burden of disease associated with dementia in older people (85+ years).

There is a consensus among stakeholder groups that inefficiencies in dementia care should be minimised, and that meaningful outcome data that are consistent, comparable, and feasible, are crucial to improving quality of care (4,5). Core Outcome Sets (COS) are designed to provide consistency and comparability, though they are not yet implemented and evaluated at a policy level or as a form of national benchmarking, and the feasibility of this approach is not currently known. This research aims to bridge this gap by evaluating existing core outcomes used for monitoring quality of routine dementia care around the world, and adapting these to fit routine data collection within the Australian care setting.

Changing long-term data collection in industry (i.e. outside the controlled trials environment) poses challenges relating to feasibility and consensus. It is not always possible or economically viable to change the type or amount of routinely collected data. To maximise the feasibility of collecting and reporting core outcome sets, this research will engage with industry partners to identify and evaluate the measures currently routinely collected in the Australian aged care setting. The challenge of reaching consensus will be addressed by engaging multiple stakeholders with varying perspectives on the importance of different outcome measures using a co-designed research design guided by participatory action research principles.

Involving stakeholders in the design and development of this research will enhance the quality of research outcomes by increasing the relevance of selected measures to people affected by dementia (6). Statements from the World Health Organisation (WHO) proclaim that people with lived experience of dementia have the greatest direct interest in research outputs and are therefore central to the research process and should be involved in all stages (1). Involving people in research means conducting research with people, as opposed to 'on' or 'for' people (7). People with cognitive impairment are capable of contributing equally to decisions about their care and have the right to voice their preferences extending beyond individual care planning into policy and research priorities (8). The involvement of people affected by dementia in the identification of core outcomes to measure the quality of their care creates a balance between individual autonomy and collective community values that facilitates equitable access to health and care services (6,9,10).

For peer review only

The objective of this research is to develop, test and provide implementation recommendations for a core outcome set applicable to routine care for people living with dementia. The core outcome set will be derived from an international literature search, current industry routine data collection, and outcomes considered important by a diverse range of stakeholders. Consensus will be sought through a variety of participatory action research activities including meetings, discussion boards, and consensus surveys.

The COS developed during this research will describe a minimum level of routinely collected information that is critical to the determination of quality of care provided to people living with a diagnosis of dementia. The COS will be trialled in aged care settings that already routinely collect data and regularly deliver mandatory reports to government. Establishing feasibility here provides the potential to disseminate the COS to additional care settings with an interest in improving quality of care for people living with a diagnosis of dementia.

At a national level, this research – the Core Outcome Measures for Improving Care (COM-IC) project - is aligned with several recommendations made in 2021 by the Royal Commission into Aged care Quality and Safety. Specifically, the project aligns with: requirements for ongoing research into the development and use of an evidence base for quality indicators; publication and guidance on data use; methodology for benchmarking; setting improvement targets; and public reporting of performance against measurable data (11).

The aim of this protocol is to outline the research plan for COM-IC. This encompasses the development of a process for involving people living with dementia in decisions about the measurement of dementia care and using participatory methods to facilitate working partnerships with stakeholders, enabling timely translation of evidence-based research into practice.

Methods and analysis

Study setting

The majority of people living with dementia seeking care access services in the aged care setting, either in residential care, or in community-based aged care programs (8,12). These organisations routinely collect and report outcome measures, meaning they are well positioned to accept and implement recommendations for core outcome measurement. As such, this research is positioned in the aged care setting, incorporating both residential aged care and home care services.

Methods

The research design is a multi-component study that applies the Alignment – Harmonisation – Results (AHR) framework (13) that preferences stakeholder engagement through a Participatory Action Research (PAR) methodology (14). Specific methods for sourcing, evaluating, and ultimately recommending outcomes are unknown at the protocol stage, as they will be co-designed as a collaboration between the international investigators and the stakeholder reference group. It is anticipated that relevant outcomes will be identified and presented to stakeholders through the international scoping review. This structure will then be adapted and expanded based on additional information such as outcomes collected by registries, by industry, and outcomes that are considered important by stakeholders.

Participatory Action Research (PAR)

PAR is an approach to research that emphasises the engagement and empowerment of research participants, sharing the responsibility for decision-making with those most impacted by the research at each stage of the research process, including design, methods, data collection, analysis, and reporting (15). From inception, the COM-IC project has involved people with lived experience of

For peer review only

dementia, as consultation with this community is fundamentally critical to generating research that meets the needs of healthcare recipients. By design, this approach limits the ability to plan and define specific methodologies for the life of the project, as the design requires stakeholder input. Consequently, specific and granular details are not able to be described at the protocol stage, instead they develop organically through collaborative processes and are incorporated into the research protocol and ethics approval via amendments.

Alignment – Harmonisation – Results Framework

The AHR Framework is a model developed to improve resource allocation in global aid, a system with similar complexities to health care quality (13). The COM-IC project adapts this framework to fit the dementia care context (Figure 1).

Figure 1 AHR Framework

Alignment

Alignment is achieved through reaching agreement on a suite of standardised (core) outcomes that define a baseline or minimum standard for all dementia-related interventions and care delivery models in Australia. As there is no consistent approach to quality and outcomes measurement for dementia care across all settings, reaching agreement first involves stakeholders and international research partners collating existing quality measures for people living with dementia, including those routinely collected by our industry partners. This list will be compiled from various sources across research, industry, and stakeholder groups.

Information on existing data collection will be included alongside the results of a scoping review of international core outcome sets for dementia, using the COMET (Core Outcome Measures in Effectiveness Trials) database, including their application and use (3,16,17), commonly applied to trials rather than routine care. The Stakeholder Reference Group (SRG) will review the complete list of measures and develop a consensus on stakeholder preferences (18). A gap analysis will converge research, industry, and stakeholder viewpoints, providing clarity around outcome measures that are relevant to the provision of high-quality dementia care, and are feasible to implement in the Australian context (2).

Identified outcome measures with high relevance to people living with dementia will be assessed for methodological quality and relevance to the population and setting (Table 1).

Table 1 Criteria for inclusion of outcome measures

Criterion	Description
**Clarity**	An outcome measure should be specific, stating clearly what is being measured, how it is measured and its interpretation
**Validity**	The outcome measure should measure what it intends to measure (i.e., it discriminates between good and bad performance), is not subject to large variation due to random changes in small numbers of events, and minimises potential bias
**Measurability and timeliness**	The performance aspect should be measurable and quantifiable
**Consistency over time**	The outcome measure should measure quality aspects consistently over time and in a timely manner
**Added value**	The outcome measure should capture elements of performance not captured elsewhere

For peer review only

**Measure of value (benefit)**	The outcome measure should capture aspects of performance proven to be valued by users and providers of services
**Attribution and granularity**	The outcome measure should measure aspects of performance that can be attributed to healthcare or aged care settings, and that are subject to healthcare or aged care system control
**Ease of collection**	The outcome measure must be able to be collected within usual practice in settings where it is required to be collected or minimise additional burden on collectors if this is not possible.

Notes: modified from the Accounting for the Quality of NHS Output framework (19)

Subsequently, this information will be summarised in plain English as a decision-making tool for the Stakeholder Reference Group (SRG). A modified e-Delphi method will be applied to reach consensus, using the STARDIT tool as the primary reporting mechanism (18,20,21). The definition of consensus will be confirmed with the stakeholder reference group using discussion threads on the Loomio platform. De-identified, aggregated results from survey tools combining Likert scales and ranking exercises will be reported back to the group, with provision for discussions, questions, and answers through a combination of synchronous and asynchronous processes. Rounds will continue until the agreed definition of consensus is reached. Outcomes identified as core will be transferred to the recommendations, co-designed with stakeholders. The STARDIT approach uses structured communication techniques applied systematically to stakeholder panels through several rounds (internet-based surveys) until consensus is reached (21). Each round is summarised by the group facilitator and results reported to all participants before the next round. Rounds continue until consensus is achieved and the measures with consensus will become the core outcome measures recommended in the recommendations. It is anticipated that the group will adopt a divergent-convergent approach to building a list of outcomes. Each list from identified sources will be considered for inclusion and evaluated against the NHS Quality Output Framework.

This approach is designed to minimise the likelihood of missing data during the consensus-building process. If missing data is identified, the primary course of action will be to afford the participant further opportunity to offer their opinion.

Harmonisation

Harmonisation refers to the generation of a standardised set of recommendations for core outcome measures in dementia care. Following the GRADE method, these recommendations will include methods to appraise, monitor and evaluate interventions and models of care (e.g. functional decline, economic evaluation, societal benefit cost analyses), or methods to measure (key) performance indicators at the organisation and system levels (e.g. composite indicators for quality of care and operational efficiency at the organisation level, or equality/disparity of access and utilisation at the sector level) (22). They will also include guidance for the selection of core outcomes for well-defined target populations (e.g. people with younger onset dementia), types of interventions, care settings and sectors. The recommendations will be accompanied by a recommended implementation plan that addresses potential barriers to implementation identified through the Alignment phase, a clear timeline, and stakeholder responsibilities.

A Recommendation Development Group (RDG) will be established that comprises members of the research team, SRG, and representatives from the Australian Department of Health and Aged Care.

The Harmonisation phase will include preparation for the feasibility study (to be conducted in the Results phase). Outcomes of the gap analysis will be used to identify data that are not currently collected by partner organisations. The feasibility of data collection will be determined through focus

For peer review only

groups and interviews conducted internally with partner organisations' staff, care recipients and family carers to understand whether additional data collection is acceptable, and the most practical methods of collection. An adoption timeline will be developed, and a sub-set of outcome measures identified that are deemed feasible for trial implementation within the partner organisation.

Results

The results phase will comprise a feasibility study with industry partners that focuses on the translation of research into practice. Data relevant to core outcome measures will be extracted from partner organisations' databases pre- and post-study and evaluated to determine both the functionality and usability of the outcome measures. A qualitative evaluation of the implementation experience will determine the practicality and sustainability of the recommendations and implementation plan developed in the harmonisation phase.

Representatives from two large, Australian aged-care providers are embedded in the COM-IC investigator team. They will support the overall project by providing information on current data collection during the alignment phase, and support their organisations to implement the core outcome recommendations in the results phase. Each partner organisation will trial the implementation of recommended measures that can be collected within existing data collection systems for six months. This may include modification of existing instruments to collect additional information relating to new recommended indicators.

The recommendations and implementation plan will be co-evaluated by the SRG and investigation team, and revised based on outcomes of the feasibility study (23). Any identified corrections will be made to produce a final set of recommendations.

PPI Statement (Stakeholder Engagement)

Methods used to involve people affected by dementia in this project have been informed by the participatory action research (PAR) paradigm (24). This approach was selected following consultation with people with lived experience of dementia. The PAR paradigm guides stakeholder involvement alongside the AHR framework by embedding participation and action in the research process. Involvement refers to using participatory methods such as workshops, focus groups, Delphi panels, surveys, policy exercises, and mutual learning activities to facilitate inclusion of stakeholders with previously limited involvement in research activities like development, design, conduct, analysis, interpretation, dissemination, implementation, and evaluation (14,25).

Dynamic Learning Needs Assessment

The premise of PAR and co-design is that stakeholders are experts of their lived experience, while researchers are experts in a particular field of study, and each brings specific knowledge to the research process that produces a more nuanced response to a research question. As the application of PAR methodology for people living with dementia is relatively recent and rare, particularly for the length of time stakeholders are asked to commit to COM-IC, we intend to evaluate the effectiveness of participatory action research methods, and investigate changes that participation has on attitudes, beliefs, and understanding of co-design research through a dynamic learning needs assessment. The assessment is a co-designed survey tool administered quarterly using the Qualtrics platform. Questions directly assess learning and support strengths and weaknesses, attitudes to research and beliefs over who should be involved in this research. Responses are linked, so changes to responses can be tracked over the duration of the study, allowing investigators to describe the effect of participatory action research on both researchers and participants with lived experience.

Population and participation

For peer review only

The potential involvement of people with cognitive impairment carries additional ethical considerations (26,27). Involvement of people living with dementia is actively encouraged and supported to maximise inclusivity, with levels of influence and support tailored to individual capabilities. People with a lived experience of dementia will be involved with all aspects of research design and implementation. The establishment and operation of the Stakeholder Reference Group will be recorded and analysed to demonstrate the changes in the wider research team and the influence of PAR on final research recommendations. Evidence of personal development over the project will be collected through the dynamic learning needs assessment, a quarterly survey completed by all members of the SRG, investigator team and project team. Evidence of impact will be collected through documentation of meetings and correspondence between consumer participants and the investigation team. Methods of involving people, reported personal development, and other impacts and outcomes will be reported using STARDIT (20).

Stakeholder Reference Group (SRG)

The SRG will guide participatory action throughout the AHR phases. The main role of the SRG is to ensure the measures including in the COM-IC recommendations are meaningful and relevant to people living with dementia while being practical and sustainable for care providers. The SRG will include a diverse range of stakeholders that reflects the heterogeneity of different groups affected by dementia.

Eligibility

The SRG will have between eight and 15 members, including at least four people who have either had an experience of using dementia care and services in the last 10 years, or have been affected by dementia (including parents, loved ones and care partners). The SRG will also include at least one person who identifies as Aboriginal or Torres Strait Islander , at least two people with professional dementia research or health professional experience, and at least two people with a professional experience of working in dementia care. SRG members will be expected to speak from their personal experience and, where appropriate, collate the views of people with an experience of dementia. SRG members will require a working knowledge of basic computer software – training will be provided as required to support participation.

Recruitment and participation

Members of the SRG will be recruited through an online expression of interest, promoted through the professional and industry networks of the research team and established dementia support networks. Expressions of interest will be reviewed by a subset of the research team, including two consumer investigators. Members will be selected based on their experience, ability to represent a diverse population, and the time they are able to commit to SRG activities. Once informed consent is received from all SRG members, and any competing or conflicting interests have been declared and publicly reported, SRG activities will commence. Potential SRG members must have sufficient cognitive capacity at the time of their recruitment to provide informed consent – individuals incapable of providing consent will not be invited to join the SRG. Potential SRG members with a diagnosis of dementia or cognitive impairment will be contacted by a member of the investigator team with experience working with people living with dementia, who will discuss what SRG participation involves, address any concerns, and confirm informed consent to participate. Acknowledging that dementia is a degenerative condition with variable rates of decline in cognitive capacity, consent will be confirmed and assented at each project activity.

Working groups

Task-specific working groups may be established to seek broader input and experiences at some stages of the COM-IC project. An online expression of interest process similar to that for the SRG will

For peer review only

be used to identify participants for each working group. Depending on the activity's objectives, eligibility criteria may include specific research experience, experience of living with dementia and/or experience of providing informal or formal care for a person living with dementia.

Outcome measures

Primary outcome

- The recommended core outcome set is appropriate to the Australian aged-care setting: Clarity – what and how outcomes are measured is understood by people with data collection and reporting responsibilities
- Validity – Outcome measures are sensitive to changes in determinants of quality care provision in routine care settings
- Measurability – Outcome measures are easily integrated into existing data collection routines and are elements that can be reliably quantified.
- Consistency – Outcome measures are collected and reported the same way across different settings and organisational structures.
- Value – Outcome measures are comprehensive and have a low administrative burden

Secondary outcomes

Secondary outcomes will include:

- Effectiveness of the PAR methodology:
  - SRG membership is sustained
  - SRG members contribute at each stage of the research program
  - SRG are able to communicate ideas and decisions to the investigators
  - SRG endorse the final core outcome recommendations
- Changes in stakeholder and researcher capacity, perspectives and beliefs measured using a dynamic Learning Needs Analysis.
  - Changes in attitudes
  - Changes in learning and support needs
  - Changes in beliefs
  - Changes in skills

Data collection

Data will be collected by members of the investigation team and industry partners. Data collected by the investigator team will include information about the PAR process, SRG and working group participants and activities. Data collected by industry partners will include information related to the feasibility study (outcome measures, implementation, acceptability). All data collection will be electronic.

Data management

Data collected for the COM-IC Project will be managed by the project team at The University of Queensland. Data will be protected by multi-factor authentication across two platforms; Microsoft Teams, and the secure research data manager (RDM) developed by the University of Queensland and cited as an approach to good research data management practice (28). All data will be regularly backed-up as part of the RDM. Password protection and multi-factor authorisation will be used to ensure that only approved members of the research and project teams will be able to access data. Any identifiable data will be de-identified prior to any public dissemination. Any information collected by partner organisations will be securely stored under password and two-factor authorisation until it can be de-identified and transferred to The University of Queensland.

Quality control

For peer review only

The secretariat will routinely evaluate data collection and storage and maintain the RDM record. Any adjustments required will be communicated with the group and regular data reports presented at quarterly meetings.

Analysis plan

The final data analysis plan will be co-developed with the SRG and is dependent on the outcome measures identified during recommendation development. Data analysis will be jointly conducted by the research team, SRG, and working groups, using agreed methods appropriate to data type. Stakeholders will be involved in data analysis and checking, developing conclusions, and reporting outcomes. Any conclusions from the analysis will be collectively agreed and disseminated in a publicly accessible way, including open-access, peer-reviewed publications. Analysis will focus on determining the feasibility of implementing core outcome measures for dementia care, the impact and effectiveness of PAR, and the data collected by the core outcomes set.

Feasibility analysis will be jointly conducted by the investigator team and a feasibility working group that includes members of the SRG. The methodology includes tools such as surveys and interviews to measure change readiness, beliefs, attitudes, and behaviours of system actors that influence implementation, measures of fidelity, and time and cost of recommendation implementation.

Impact and effectiveness of PAR will be analysed using data on *level of engagement* (e.g. meeting attendance, participation in activities, contributions, and roles undertaken), *change in research capacity* (based on the dynamic learning needs assessment), and *ability to achieve consensus (*using tools such as a modified e-Delphi method). The overall PAR methodology will be reported using STARDIT (20). SRG and working group impact on the COM-IC Project will be evaluated using qualitative methods, including data mapping and familiarisation; transcription; coding; searching for themes; reviewing themes with study team members; labelling and summarising themes; and reporting the findings. In order to enhance validity of the analysis, two study team members will independently analyse the data thematically, with the analysis then checked for validity ('member checked') by a third study team member (29–31). STARDIT-PM categories will be used to organise data into pre-defined 'super-categories' that allow consistent comparison with other initiatives reporting with STARDIT (20).

Core outcome set data analysis methods will be co-developed by the SRG and investigator team and will incorporate suitable quantitative methods, e.g. descriptive statistics such as mean, standard deviation, interquartile range and baseline comparisons.

Discussion

Improving care for people living with dementia requires timely and reliable diagnosis, empowerment, and re-enablement of each individual in participating and planning their care, speedy translation of clinical evidence into practice and policy, and effective co-ordination of care provision across settings. Rapid translation of evidence into research remains a challenge due to the complexity of healthcare and social care services, limited funding resources for services, and lack of consensus on what constitutes "good value care" between individuals living with dementia, service providers and funders.

Rapid translation of COM-IC project findings is feasible as many outcome measures are already embedded in existing information systems and used as key performance indicators or accreditation standards by aged care providers (5,32), and/or used in dementia research (trials, cohort or implementation studies of innovative care delivery models) (4,17,33–35).

The outcome of this project will be recommendations for a set of core outcome measures for routine care of people living with dementia that encompass both consumer preferences and international

For peer review only

evidence-based care and are tested and suitable for use in the aged care setting for people living with dementia.

There are some limitations that may impact the generalisability of our study and complicate translation of our research into practice. In the first instance, there are numerous sources of outcome measures that may be difficult to condense sufficiently and maintain the coverage required for the breadth and complexity of routine care provision. As there has not been consistency in outcome measurement for routine care, it is not possible to compare whether the outcome set developed is superior to any other. For the feasibility study, the time for data collection may not be adequate to demonstrate sensitivity of the outcome measures to changes in care provision, as some indicators take a longer period of time to show change. Finally, our stakeholder reference group is designed to be representative of large segments of the community, but in itself is comprised of individuals that may not necessarily hold the beliefs and values of the entire community they represent.

Ethics and dissemination

Research ethics approval

This study has received ethical approval from the University of Queensland (HREC 2021/HE001932) and has received approval in-kind from partner organisations. Any modifications to the protocol that may impact on the conduct of the study, including changes of study objectives, study design, study population, sample sizes, study procedures, or significant administrative aspects will be formalised as a protocol amendment, subject to HREC approval and subsequent notifications to authorities in accordance with local regulations.

Additional considerations for potential cognitive impairment

Accommodations for people with varying levels of cognitive ability, including the potential for deterioration over the course of the study, have been discussed. In the '*Blueprint for dementia research*', the WHO highlights the importance of involving people with lived experience at all stages of the research process, noting it is part of their rights under United Nations Convention on the Rights of Persons with Disabilities and other human rights instruments (1). Involvement ensures the most efficient translation of research into practice, producing outcomes that are relevant to the people they most affect.

In line with the WHO blueprint, additional consideration has been given for accessibility, data-sharing, capacity building, strengthening support networks, utilising existing advocacy and support structures, use of technology, and sharing knowledge (1). Information is packaged in different ways and able to be tailored to the level of comprehension of the individual. Ongoing dynamic learning needs assessment monitors progress and motivation within the project team. Outcomes of the research will be published and distributed widely along familiar routes including academic and industry publications, symposia, and conference presentations. Audiences are diverse, including people with lived experience, clinicians, care professionals, aged care providers, advocacy groups and academics. Assent processes are used to confirm ongoing understanding of the research process. Representation of people with lived experience at every stage is factored into the design, as well as strategies for addressing any discomfort or potential harm as it is identified. Once the SRG is established, members will distribute findings through their networks and recruit working-group members from the dementia community. Any findings will be distributed through SRG networks, meaning the broader dementia community have rapid access to results and have a familiar conduit to provide feedback in real-time to the research team over the course of the project.

For peer review only

Consent and assent

Prior to commencing any research activities, all people included in the study will receive written participant information sheet and will sign a consent form. They will have the opportunity to ask any questions. For people with potential cognitive impairment, pictorial versions have been developed. As dementia is a progressive condition with uncertain prognosis, assent is requested prior to any recording of information. Participants are not required to participate against their will and are free to withdraw at any time.

Confidentiality

All potentially sensitive information will be stored securely on The University of Queensland's research data management system (RDM). This system complies with all international data safety requirements. All project-related data will be stored in a durable format alongside project metadata, which will be regularly backed up by secure ITS servers. Data is accessible only to individuals listed on the project record and are accessible only via institutional usernames and passwords. Information published in reports, academic papers, and presentations, will be de-identified. There is a small risk of identification through familiarity explained to participants in the consent process.

Dissemination of research findings

Results will be disseminated through networks established over the project, and in accordance with both the publication schedule and STARDIT reports.

Data Statement

Full access to outputs will be made available through UQ eSpace, an open access repository hosted by the University of Queensland.

Funding statement

This research is funded by a Medical Research Future Fund (Dementia Ageing and Aged Care Mission) targeted grant, awarded in 2021 (2007650). The funder has no role in study design, data collection, analysis or publications decisions.

Competing interests

Jack Nunn is the volunteer Director of the registered charity 'Science for All,' and is paid as an individual by the charity for his work on this MRFF funded project by written agreement. Science for All will receive funding through the COM-IC MRFF grant to conduct elements of the research program. All funding is reported annually through the Australian Charities and No-for-profits Commission (ACNC).

For peer review only

Authors Contributions

Concept: Developed through collaboration and communication between TC, KHN, LG, LF, PW, SD, CC, TM, JN, DT, OA, AW, JL, JQ, GP and SK.

Design: All authors contributed design ideas, finalised by TC, AW, DK.

Component leads: Overall project: TC; International review of core outcome measures led by AK; recommendation development led by TC and SK; Industry analysis led by TM and AM, supported by CC and JL; Gap analysis conducted by THDT, assisted by DK, AK, TM and AM. Recommendation development: SK and TC; Feasibility study; JL, CC, supported by AM and TM. Stakeholder Reference Group: JN supported by DK.

Industry Contributions: LF, OA represent hospital care; CC, TM, JL, AM represent residential aged care and home care for older people; CC, SK, LF represent advocacy and dementia support networks. DT, KHN, AW, SD, PW give expertise and insight on core outcome measures and their applications.

Stakeholder Reference Group establishment and analysis; JN, DK, AW, TC.

Writing: TC, DK, AW.

Editing: All authors assist with editing and proofreading manuscript for final submission.

For peer review only

References

1. World Health Organization (WHO). A blueprint for dementia research [Internet]. Internet; 2022. Available from: https://www.who.int/publications/i/item/9789240058248

2. Lord K, Beresford-Dent J, Rapaport P, Burton A, Leverton M, Walters K, et al. Developing the New Interventions for independence in Dementia Study (NIDUS) theoretical model for supporting people to live well with dementia at home for longer: a systematic review of theoretical models and Randomised Controlled Trial evidence. Soc Psychiatry Psychiatr Epidemiol. 2020;55(1):1–14.

3. Williamson PR, Altman DG, Bagley H, Barnes KL, Blazeby JM, Brookes ST, et al. The COMET Handbook: version 1.0. Trials. 2017 Jun;18(S3):280.

4. Dodd S, Harman N, Taske N, Minchin M, Tan T, Williamson PR. Core outcome sets through the healthcare ecosystem: the case of type 2 diabetes mellitus. Trials. 2020 Jun 25;21(1):570.

5. Tran A, Nguyen KH, Gray L, Comans T. A Systematic Literature Review of Efficiency Measurement in Nursing Homes. Int J Environ Res Public Health. 2019 Jan;16(12):2186.

6. Bethell J, Pringle D, Chambers LW, Cohen C, Commisso E, Cowan K, et al. Patient and Public Involvement in Identifying Dementia Research Priorities. J Am Geriatr Soc. 2018;66(8):1608–12.

7. Briefing notes for researchers - public involvement in NHS, health and social care research [Internet] [Internet]. [cited 2024 May 22]. Available from: Briefing notes for researchers - public involvement in NHS, health and social care research

8. Waller M, Mishra GD, Dobson AJ. Estimating the prevalence of dementia using multiple linked administrative health records and capture-recapture methodology. Emerg Themes Epidemiol. 2017;14(1).

9. Kelly S, Lafortune L, Hart N, Cowan K, Fenton M, Brayne C, et al. Dementia priority setting partnership with the James Lind Alliance: using patient and public involvement and the evidence base to inform the research agenda. Age Ageing. 2015 Nov 1;44(6):985–93.

10. Ratcliffe J, Hutchinson C, Milte R, Nguyen KH, Welch A, Caporale T, et al. How do people with dementia and family carers value dementia-specific quality of life states? An explorative “Think Aloud” study. Australas J Ageing. 2019;38(S2):75–82.

11. Royal Commission into Aged Care Quality and Safety [Internet]. 2021 [cited 2023 Apr 24]. Final Report - Volume 1: Summary and Recommendations. Available from: https://agedcare.royalcommission.gov.au/publications/final-report-volume-1

12. Rozsa M, Ford A, Flicker L. Assessing people with dementia: the role of the aged care assessment team and memory clinics. Intern Med J. 2016;46(9):1081–8.

13. OECD World Bank. Harmonisation, Alignment, Results [Internet]. The World Bank; 2007. Available from: https://doi.org/10.1596/978-9-2640-3673-4

14. NIHR. What is Patient and Public Involvement and Engagement? [Internet]. [cited 2023 Jan 17]. Available from: http://www.hpruezi.nihr.ac.uk/public-involvement/

15. Vaughn LM, Jacquez F. Participatory Research Methods – Choice Points in the Research Process. J Particip Res Methods. 2020;1(1).

16. Page MJ, Moher D, Bossuyt PM, Boutron I, Hoffmann TC, Mulrow CD, et al. PRISMA 2020 explanation and elaboration: updated guidance and exemplars for reporting systematic reviews. BMJ. 2021;372:n160–n160.

For peer review only

17. COMET Initiative | Home [Internet]. [cited 2021 Feb 26]. Available from: https://www.comet-initiative.org/

18. Lee A, Davies A, Young AE. Systematic review of international Delphi surveys for core outcome set development: representation of international patients. BMJ Open. 2020;10(11):e040223–e040223.

19. Bojke C, Castelli A, Grašič K, Mason A, Street A. Accounting for the quality of NHS output. 2018 p. 62.

20. Nunn JS, Shafee T. Standardised Data on Initiatives – STARDIT: Beta Version [Internet]. OSF Preprints; 2021 [cited 2022 Nov 15]. Available from: https://osf.io/w5xj6/

21. Hasson F, Keeney S, McKenna H. Research guidelines for the Delphi survey technique. J Adv Nurs. 2000;32(4):1008–15.

22. Schünemann H, Brożek J, Guyatt G, Oxman A. GRADE Handbook for grading the quality of evidence and the strength of recommendations [Internet]. The GRADE Working Group; 2013. Available from: https://gdt.gradepro.org/app/handbook/handbook.html

23. Phillips R, Kara H. Creative Writing for Social Research. Bristol: Policy Press; 2021.

24. Pandya-Wood R, Barron DS, Elliott J. A framework for public involvement at the design stage of NHS health and social care research: Time to develop ethically conscious standards. Res Involv Engag. 2017;3(1):6–6.

25. INVOLVE. Public involvement in research: values and principles framework [Internet]. INVOLVE: Eastleigh; 2015 [cited 2021 Feb 26]. Available from: https://www.invo.org.uk/posttypepublication/public-involvement-in-researchvalues-and-principles-framework/

26. Chandra M, Harbishettar V, Sawhney H, Amanullah S. Ethical Issues in Dementia Research. Indian J Psychol Med. 2021;43(5_suppl):S25–30.

27. NHMRC. National Statement on Ethical Conduct in Human Research (2007) [Internet]. [cited 2017 Nov 10]. Available from: https://www.nhmrc.gov.au/book/national-statement-ethical-conduct-human-research

28. Directorate-General for Research and Innovation (European Commission). Turning FAIR into reality: final report and action plan from the European Commission expert group on FAIR data [Internet]. LU: Publications Office of the European Union; 2018 [cited 2023 Jan 10]. Available from: https://data.europa.eu/doi/10.2777/1524

29. Saldaña J. The coding manual for qualitative researchers. 4E ed. Thousand Oaks, California: SAGE Publishing; 2021.

30. Hyett N, Kenny A, Dickson-Swift V. Methodology or method? A critical review of qualitative case study reports. Int J Qual Stud Health Well-Being. 2014;9(1):23606–23606.

31. Guest G, MacQueen KM, Namey EE, MacQueen KM, Namey EE. Applied thematic analysis. Los Angeles: Sage Publications; 2012.

32. Comans T, Nguyen KH, Zelenyuk V, Tran A, Nguyen BT. Research Paper 9 - The cost of residential aged care [Internet]. Royal Commission into Aged Care Quality and Safety [Internet]. [cited 2021 Feb 26]. Available from: https://agedcare.royalcommission.gov.au/publications/research-paper-9-cost-residential-aged-care

For peer review only

33. Dodd S, Clarke M, Becker L, Mavergames C, Fish R, Williamson PR. A taxonomy has been developed for outcomes in medical research to help improve knowledge discovery. J Clin Epidemiol. 2018 Apr;96:84–92.

34. Vincent R, Chalmers JR, McWilliams C, Thomas KS, Dodd S, Rogers N, et al. Assessing uptake of the Harmonising Outcome Measures for Eczema (HOME) Core Outcome Set and recommended instruments. Br J Dermatol. 2020;183(3):566–8.

35. Comans TA, Nguyen KH, Ratcliffe J, Rowen D, Mulhern B. Valuing the AD-5D Dementia Utility Instrument: An Estimation of a General Population Tariff. PharmacoEconomics. 2020 Aug 1;38(8):871–81.

For peer review only

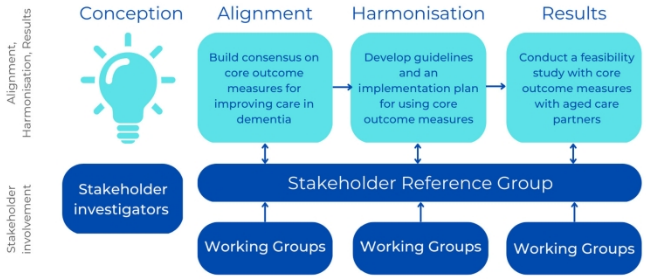

Figure 1: AHR Framework

210x297mm (300 x 300 DPI)

Item	No	Description	Page Ref initial submission
TITLE/ABSTRACT			
Title	1a	Identify in the title that the paper describes the protocol for the planned development of a COS	P3.L7
Abstract	1b	Provide a structured abstract	P4.L5-33 (as per BMJ requirements)
INTRODUCTION			
Background and objectives	2a	Describe the background and explain the rationale for developing the COS, and identify the reasons why a COS is needed and the potential barriers to its implementation	P5
	2b	Describe the specific objectives with reference to developing a COS	P5. Amended in revision
Scope	3a	Describe the health condition(s) and population(s) that will be covered by the COS	P5.L6-12
	3b	Describe the intervention(s) that will be covered by the COS	P5. Amended in revision
	3c	Describe the context of use for which the COS is to be applied	P5. Amended in revision
METHODS			
Stakeholders	4	Describe the stakeholder groups to be involved in the COS development process, the nature of and rationale for their involvement and also how the individuals will be identified; this should cover involvement both as members of the research team and as participants in the study	P8-9. Amended in revision
Information sources	5a	Describe the information sources that will be used to identify the list of outcomes. Outline the methods or reference other protocols/papers	P6.L30-37
	5b	Describe how outcomes may be dropped/combined, with reasons	P6.L39-P7.L13. Amended in revision
Consensus process	6	Describe the plans for how the consensus process will be undertaken	P7.L14-P7.L25. Amended in revision
Consensus definition	7a	Describe the consensus definition	P7.L14-P7.L25. Amended in revision
	7b	Describe the procedure for determining how outcomes will be added/combined/dropped from consideration during the consensus process	P7.L19-24. Amended in revision
ANALYSIS			
Outcome scoring/feedback	8	Describe how outcomes will be scored and summarised, describe how participants will receive feedback during the consensus process	Table 1
Missing data	9	Describe how missing data will be handled during the consensus process	Added in revision
ETHICS and DISSEMINATION			
Ethics approval/informed consent	10	Describe any plans for obtaining research ethics committee/institutional review board approval in relation to the consensus process and describe how informed consent will be obtained (if relevant)	P11.L20-P11.L22
Dissemination	11	Describe any plans to communicate the results to study participants and COS users, inclusive of methods and timing of dissemination	P11.L23-P11.L26
ADMINISTRATIVE INFORMATION			
Funders	12	Describe sources of funding, role of funders	P11.L32-P11.L36
Conflicts of interest	13	Describe any potential conflicts of interest within the study team and how they will be managed	P11.L37-P11.L44
